# Cortical Activation Patterns of Bodily Attention triggered by Acupuncture Stimulation

**DOI:** 10.1038/srep12455

**Published:** 2015-07-27

**Authors:** Won-Mo Jung, In-Seon Lee, Christian Wallraven, Yeon-Hee Ryu, Hi-Joon Park, Younbyoung Chae

**Affiliations:** 1 Acupuncture & Meridian Science Research Center, College of Korean Medicine, Kyung Hee University, Seoul, Republic of Korea; 2 Department of Internal Medicine: Psychosomatic Medicine and Psychotherapy, University Hospital Tübingen, Tübingen, Germany; 3 IMPRS for Cognitive and Systems Neuroscience, University Tübingen, Tübingen, Germany; 4 Department of Brain Cognitive Engineering, Korea University, Seoul, Republic of Korea; 5 Acupuncture, Moxibustion and Meridian Research Center, Division of Standard Research, Korea Institute of Oriental Medicine, Daejeon, Republic of Korea

## Abstract

We investigated commonalities and differences in brain responses to enhanced bodily attention around acupuncture points with and without stimulation. Fourteen participants received acupuncture needles at both PC6 and HT7 acupoints in the left hand. To enhance bodily attention to acupoints, participants responded to the locations of stimulations in a two-alternative forced choice task. Two fMRI scans were taken in a block design: session 1 labeled with manual stimulation (genuine stimulation) and session 2 labeled with electro-acupuncture (pseudo-stimulation). To compare cortical activation patterns, data were analyzed using the Freesurfer software package. Both genuine-and pseudo-stimulation resulted in brain activations in the insula, anterior cingulate cortex, secondary somatosensory cortex, superior parietal cortex, and brain deactivation in the medial prefrontal cortex, posterior cingulate cortex, inferior parietal cortex, and the parahippocampus. Genuine acupuncture stimulation exhibited greater brain activation in the posterior insula, posterior operculum and the caudal part of the anterior cingulate cortex, compared with pseudo-stimulation. We demonstrated that enhanced bodily attention triggered by genuine acupuncture stimulation can activate the salience network and deactivate the default mode network regardless of the type of stimulation. The component of enhanced attention to a certain part of the body is significant in the brain response to acupuncture stimulation.

Acupuncture is effective for the treatment of chronic pain and is therefore a reasonable referral option^1^,^2^; however, the underlying physiological mechanisms of acupuncture remain unclear. Acupuncture is a complex somatosensory stimulation including bodily attention that triggers a wide range of effects in the body^3^,^4^. A large number of neuroimaging studies have suggested that acupuncture-induced cortical activation mainly reflects the somatosensory, affective and cognitive processing of pain^5^,^6^. Recently, Bai *et al.* observed that long-term acupuncture modulated anterior-insula-associated intrinsic coherences of the limbic and brainstem regions, which are autonomic centers of the ascending and descending pathways^7^. Hui *et al.* characterized a strong deactivation of the default mode network (DMN) as a particular feature of acupuncture compared with other sensory stimuli^8^. Dhond *et al.* observed correlations between parasympathetic activation and an increase in DMN connectivity via acupuncture stimulation^9^. Changes in heart rate changes following activation of brain stem structures (including periaqueductal gray, rostral ventromedial medulla and hypothalamus) were also found following acupuncture stimulation^10^. The insular cortex is a central region implicated in many functional studies of acupuncture, and it is crucial in the interface between cognitive, homeostatic, and affective systems of the human brain, providing a switching role between stimulus-driven processing and brain regions involved in monitoring internal milieu^11^. Taken together, these results suggest that acupuncture may exert potential actions in endogenous pain modulation circuits and homeostatic control by modulating brain activity in the salient interoceptive-autonomic network and DMN.

Enhancing body ownership by viewing one’s own body or through a multi-sensory illusion experience can exert analgesic effects^12^,^13^,^14^. For example, the visual context of seeing the body modulates the experience of pain via interplay between the brain’s pain network and the posterior network for body perception^13^. Visual manipulation of the appearance of body parts was also found to be beneficial for the amelioration of chronic pain^14^. Mindfulness meditation has been shown to alleviate pain experiences, along with increased activity in the anterior insula and the anterior cingulate cortex^15^. Similarly, peripheral and central physiological responses during acupuncture were altered by modifications of body ownership, highlighting the role of body awareness in the effects of acupuncture^16^. Thus, it has been suggested that brain responses to acupuncture, especially components of accentuated bodily attention triggered by acupuncture stimulation, are highly associated with salient components of acupuncture analgesia^17^. However, despite this growing evidence, the role of bodily attention in brain responses to acupuncture stimulation has not yet been investigated to date.

The aim of this study was to investigate the response of the brain to acupuncture during manipulation of bodily attention. We aimed to determine the brain response to enhanced bodily attention both with genuine acupuncture stimulation and pseudo-stimulation. We hypothesized that enhanced bodily attention may engage brain regions, including the anterior insula and the anterior cingulate cortex, which are known for their role in the salient interoceptive-autonomic network, as well as the medial prefrontal and posterior cingulate cortices, which are involved in the DMN. Furthermore, we dissociate brain responses to external stimulation with acupuncture from bodily attention via contrast between the two conditions.

## Methods

### Participants

A total of 14 male participants (age: 22.1 ± 1.1 years) were recruited by advertisement among the students of Korea University in Seoul, Republic of Korea. The participants had no history of neurological, psychiatric, or visual disorders. Handedness was self-reported by the participants in a screening questionnaire prior to the experiment, and all participants reported that they were right-handed. Participants were prohibited from drinking alcohol or caffeine and from taking any drugs or medications on the day of the experiment. The Institutional Review Board at Korea University approved all study procedures. The experiment was performed in accordance with approved guidelines. All subjects who enrolled in this study provided written informed consent before beginning any study procedure.

### Behavioral tasks

#### Spatial two-point discrimination task

The two-point discrimination (TPD) task was used to determine the tactile sensitivity of each participant. A two-point caliper was used to apply two pressure stimuli seven separations from 0 mm (i.e., a one-point stimulus) to 60 mm at intervals of 10 mm (giving six two-point stimuli) in random order on the participant’s left hand. To ensure sustained attention, participants wore eye patches and noise-canceling head earmuffs (3M). The two points of the caliper were applied simultaneously, using the weight of the caliper alone. Following each application, participants reported whether they felt a one-point or a two-point stimulus. This procedure was repeated 10 times to yield 70 trials per participant. A general linear model was used to fit the participants’ size-change discrimination performance with robust cumulative and normal psychometric functions. The point of subjective equality (PSE) was calculated as the 50% point on the fitted psychometric functions. Curve fitting was implemented using the Psyphy R package (http://www.cran.r-project.org).

#### Two-point alternative choice task

Several methods have been used to experimentally modulate attention to somatosensory or pain stimuli in previous studies, including counting the number of stimuli^18^, rating the intensity of stimuli^9^,^19^, and discriminating a stimulus site between two adjacent body points^20^. Here, we used a modified acupuncture procedure that integrates a two-point discrimination procedure during functional magnetic resonance imaging (fMRI) scanning (see Experimental Design): tactile stimulation was applied at one of two acupoints using a 6.45 von Frey monofilament stimulator (Touch-Test Sensory Evaluators, North Coast Medical, California, USA). The task for participants was to report which site was stimulated, as well as the perceived stimulation intensity, by pressing one of four buttons (i.e., on a scale of 1 to 4). To acquaint the participants with this procedure, they received several tactile stimuli prior to the fMRI session to ensure that they could adequately perform the discrimination task. This procedure also served to familiarize them with the temporal sequence of stimuli within a series to minimize variations in cognitive components, such as expectation and anxiety.

### Experimental design

**Figure 1:**
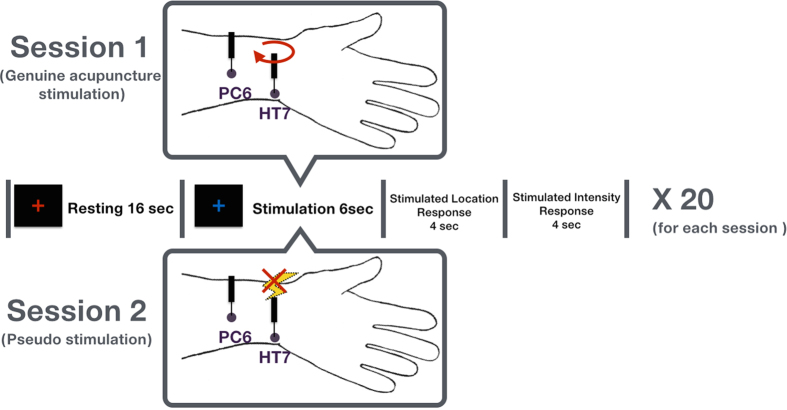
The temporal sequence of the fMRI sessions. With genuine acupuncture, stimulation was applied at the two acupoints HT7 and PC6, and participants were required to discriminate the stimulated location between PC6 and HT7. With the pseudo-stimulation session, participants were informed that electrical acupuncture stimulation would be given; however, no genuine acupuncture stimulation was given. Each session consisted of 20 trials. A red cross appeared for a resting period of 16 s, followed by a blue cross during the stimulation. Following the stimulation (either genuine or pseudo-stimulation), participants were asked to discriminate the location and to rate the intensity for 4 s.

We performed two fMRI scanning sessions: one with genuine acupuncture stimuli in which participants were told to expect manual stimulation, and another with pseudo-stimulation in which participants were told to expect electro-acupuncture (Fig. 1). The latter condition would therefore retain bodily attention at the acupuncture site, but the participant would not actually receive acupuncture stimulation. To enhance the credibility of the tactile perception in the pseudo-stimulation condition, we fixed the order of conditions such that the genuine acupuncture stimulation condition (session 1) always came first, followed by the pseudo-stimulation condition (session 2). Importantly, the participants were informed that the first session would include standard acupuncture stimulation, whereas the second session would include electrical acupuncture stimulation. Although the acupuncture needles were inserted during the second session as well, stimulation was not given during this session, resulting in “pseudo-stimulation”.

Each session of the experiment consisted of 20 trials. In the first session, each discrimination trial started with a 16-second rest period, during which participants were told to fixate on a red cross, followed by a 6-second acupuncture stimulation period at either the PC6 or the HT7 acupoint. During this period, a blue fixation cross was shown as the visual stimulus. The order of acupoints within each session was counter-balanced and randomized across participants. Following the stimulus, participants were required to discriminate the stimulated location between PC6 and HT7 using two-alternative forced choice (2AFC) task and then to rate the intensity of the sensation by pressing one of the four buttons on a four-button MRI compatible button box (Current Design™), which was held in the right hand during the entire session. Each rating screen appeared for 4 seconds, and participants were instructed to make their decision before the end of this period. The overall time for each trial was 30 seconds.

The task and protocol of the second session were designed to parallel the first session. Participants were informed that they would receive electrical acupuncture stimulation during the blue cross stimulus (pseudo-stimulation). Again, the only physical difference between the two conditions was that no external physical stimulation was applied. For the fMRI acquisition series, participants’ discrimination responses were recorded using the Matlab toolbox Psychtoolbox. The data were processed using customized programs within the R software package. Error rates were examined to identify the effects of acupoints on the reported sensation intensity. Two participants among 14 participants were excluded in the pseudo-stimulation session as their responding rate was less than 80%. During the debriefing, they reported that the stimulation intensity in some trials was too weak to discriminate the locations of stimulation. However, there was no participant who expressed suspicions about the administration of electrical stimulation in the pseudo-stimulation session.

### Acupuncture stimulation

Acupuncture stimulation was applied at the two acupoints HT7 and PC6 on the left hand by a licensed and experienced Doctor of Korean Medicine. Non-magnetic titanium sterile acupuncture needles 40-mm-long and 0.20 mm in diameter (DongBang Acupuncture Inc.; Boryeoung, Republic of Korea) were used. All stimulations were administered according to the beat of a 1-Hz metronome transmitted via earphones.

### fMRI data acquisition

The fMRI scans were acquired using a Magnetom Trio 3T scanner (Siemens, Erlangen, Germany) using echo planar imaging (EPI) with a 64 × 64 matrix (TE = 30 ms and TR = 2000 ms) across 37 slices, each 4 mm in thickness. To minimize movement artifacts, the head of each subject was fixed using a head holder, and all images were acquired by a well-trained professional operator. Each scan session consisted of 300 volumes of the whole brain in 37 axial slices (TR = 2000 ms, TE = 30 ms, flip angle = 90°, field of view = 240 × 240 mm^2^, voxel size = 3.8 × 3.8 × 4.0 mm^3^). As an anatomical reference, a three-dimensional (3D) T1-weighted magnetization-prepared rapid gradient echo (MPRAGE) image dataset was acquired using the following parameters: TR = 2000 ms, TE = 2.37 ms, flip angle = 9°, field of view = 240 × 240 mm^2^, voxel size = 0.9 × 0.9 × 1.0 mm^3^, and 192 slices.

### fMRI data analysis

The functional imaging data were preprocessed and analyzed using Nipype^21^, which is a pipeline platform that joins software packages, including SPM8 (Welcome Department of Cognitive Neurology, London, United Kingdom, http://www.fil.ion.ucl.ac.uk/spm), Freesurfer (http://surfer.nmr. mgh.harvard.edu), and FSL (http://www.fmrib.ox.ac.uk/fsl/index.html) into a single workflow. In the preprocessing stage, a rigid-body transformation was used to realign the functional images to the mean EPI image, correcting for subject head movement and for slice timing. Outliers with movements >1 mm or with an intensity Z-threshold >3 standard deviations (SDs) from the mean were removed from the data using an artifact detection algorithm (http://www.nitrc.org/projects/artifact_detect). Surface-based analysis can provide more specific results for cortical structures based on the individually-reconstructed anatomical structure of cortical surfaces^22^. Moreover, this analysis is potentially more sensitive because its search domain is small (no white matter and no cerebrospinal fluid)^23^. Cortical surface models can also simplify data visualization by revealing the pattern of activation throughout the whole cortex in one view. For the surfaced-based analysis, Freesurfer was used to segment each anatomical volume into gray and white matter structures and to perform cortical surface reconstruction. The mean functional image generated by realignment was registered to each subject’s reconstructed structural MRI data^24^. The functional images were smoothed on the cortical surface using a Gaussian filter with a full-width at half-maximum (FWHM) of 4 mm. In addition, a whole-brain analysis was conducted that also encompassed subcortical regions and the cerebellum using AFNI software (NIMH, USA); the detailed methods and the resulting activation patterns are reported in Supplementary Table S1.

For each stimulus, a boxcar function was used to represent the onset of each event. These time series were then convolved with a canonical hemodynamic response function (HRF) to generate a simulated blood-oxygen-level dependent (BOLD) response. A standard hierarchical group model approach was used to fit the simulated response to scan time-courses^25^. Contrast images were generated for each subject. Conditions were treated as fixed effects. A “summary statistics” procedure was used to model the group effects, performing one-sample *t*-tests across the individual contrast images. The model was applied with a *t*-value threshold of 2 and a cluster-threshold correction for multiple comparisons using a Monte Carlo simulation with 10,000 iterations, resulting in *p* < 0.05^26^.

To formally test whether any voxels were significantly activated (stimulation > baseline) during both conditions (genuine and pseudo), we tested the conjunction map under the “conjunction null” hypothesis. For this, we calculated a minimum Z-statistic image between two contrast images (genuine and pseudo) generated during the analysis of main effects of two conditions as a conjunction map of each individual. For the group-level statistical map of the conjunction analysis, a one sample *t*-test was performed across individual conjunction maps. This conjunction approach asks for the common neural correlates of both genuine acupuncture stimulation and pseudo-stimulation. A group-level statistical map of the difference comparing genuine- and pseudo-stimulation was created by performing paired t-tests between the two contrasts (genuine and pseudo) generated during the main-effect analysis of the two conditions across individuals. This difference map helps to reveal neural correlates of afferent signal processing. The resulting statistical parametric maps were corrected for multiple comparisons using a Monte Carlo simulation (with 10,000 iterations) using a cluster-wise probability threshold. Significant clusters were retained with a cluster-wise probability threshold of 0.01.

An additional analysis was run to show correlations across individuals between sensitivity to external stimuli and inter-subject variability in differences of fMRI responses between genuine- and pseudo-stimulation. The map of covariates was created by performing an analysis of covariance (ANCOVA) on individual contrasts calculated by subtracting the contrast of the pseudo condition from the contrast of the genuine condition; the inverse of the demeaned PSE value in the two-point discrimination task was used as the covariate of interest. The covariate map was thresholded at p < 0.001 (Z > 3, uncorrected). To visualize the cortical activation map, statistical parametric maps were overlaid on a high resolution surface template, a default averaged template with high resolution (163842 vertices; 327680 faces) provided by Freesurfer.

## Results

### Behavioral results

Participants estimated their stimulated acupoint and intensity of stimulation on a scale of 1 to 4. Of these estimations, 98.6% were correct in the genuine acupuncture session; however, in session 2 (pseudo-simulation), the response frequency of each point was distributed equally between the two acupoints (PC6: 9.2 ± 2.9 vs. HT7: 8.8 ± 3.3, *t* *=* −0.3661, *p* = 0.7202). In the genuine acupuncture session, the intensity of the PC6 stimulation was not significantly different from that of the HT7 stimulation (2.5 ± 0.9 vs. 2.7 ± 0.9, *t* *=* 0.7016, *p* = 0.4953). The same was observed for session 2 (1.2 ± 0.6 vs. 1.3 ± 0.5, *t* = 0.7506, *p* = 0.4663). However, differences in the intensities were observed between the two sessions (2.6 ± 0.6 vs. 1.2 ± 0.3 *t* = 11.1935, *p* < 0.001, Table 1).

### BOLD signal response to genuine acupuncture and pseudo-stimulation

**Figure 2:**
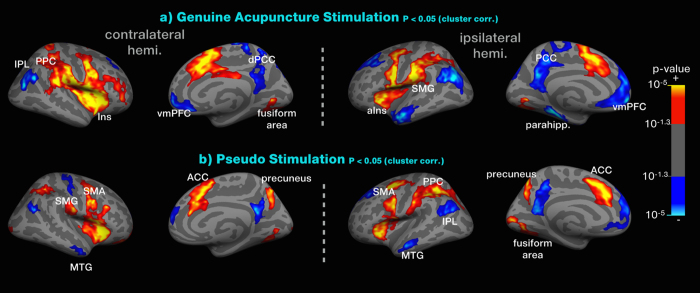
Cortical activation related to genuine acupuncture and pseudo-stimulation. The genuine stimulation produced significant activation of the insula, operculum, SMA, SI, SII, SMG, PPC, ACC and fusiform areas, whereas the anterior insula, SMA, SMG, PPC, ACC, fusiform area and precuneus were activated in response to pseudo-stimulation. Significant decreases in the DMN, including the vmPFC, PCC, IPL, MTG and parahippocampus, were observed in both conditions.

We found significant BOLD signal increases in response to genuine acupuncture stimulation in the following areas: bilateral large cluster including insula, operculum (the contralateral hemisphere includes more posterior insula and extending to inferior frontal gyrus), supplementary motor area (SMA), primary somatosensory cortex (SI), secondary somatosensory cortex (SII), supramarginal gyrus (SMG) and posterior parietal cortex (PPC), bilateral anterior cingulate cortex (ACC) and fusiform area (*p* < 0.05, cluster correction). We also found significant decreases in the DMN, including ventromedial prefrontal cortex (vmPFC), posterior cingulate cortex (PCC), inferior parietal lobe (IPL), medial temporal gyrus (MTG) and parahippocampus. Table 2 lists a summary of these data Fig. 2 shows the results of the BOLD images with genuine acupuncture stimulation and with pseudo-stimulation.

With pseudo-stimulation, we found the BOLD signal to increase in the following areas: the bilateral anterior insula (aIns), SMA, SMG, PPC, ACC, fusiform area as well as the precuneus. We also found significant decreases in the DMN, including the vmPFC, PCC, IPL, MTG and parahippocampus. Table 3 lists a summary of these data.

### Common brain responses to genuine acupuncture and pseudo-stimulation

**Figure 3:**
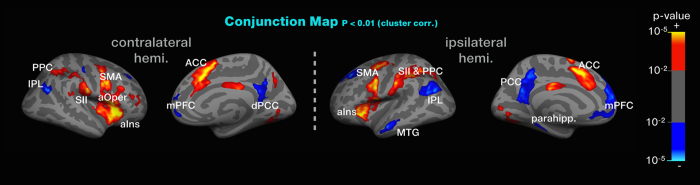
Cortical regions exhibited greater activity during both genuine acupuncture and pseudo-stimulation. The thresholds for statistical significance in this conjunction analyses were *p* < 0.01, cluster-threshold corrected. Significant activation in the aIns, anterior operculum (aOper), SMA, SMG, PPC, ACC, fusiform area and significant deactivation in the DMN was observed.

To identify common brain responses to both genuine acupuncture and pseudo-stimulation, we carried out a conjunction analysis on the two main effects. This analysis revealed significant activation of the aIns, anterior operculum (aOper), SMA, SMG, PPC, ACC, and fusiform area and significant deactivation of the DMN, suggesting that these brain regions represent top-down components during genuine acupuncture and are likely to contain aspects of bodily attention. Table 4 lists a summary of the fMRI conjunction map data for genuine acupuncture stimulation and pseudo-stimulation. Fig. 3 shows the fMRI conjunction maps.

### Differential brain responses to genuine acupuncture and pseudo-stimulation

**Figure 4:**
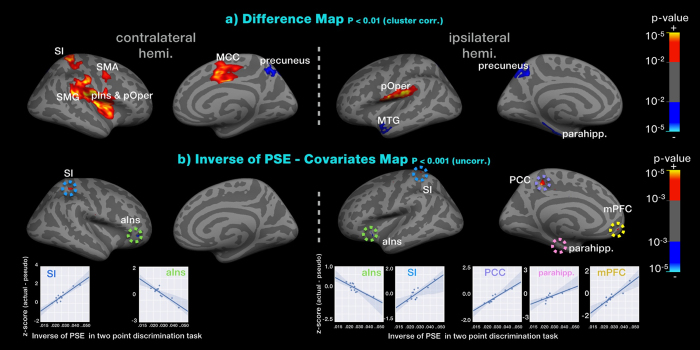
(**a**) fMRI group difference maps between genuine acupuncture and pseudo-stimulation. Genuine acupuncture induced greater activation in the pIns, SMG, SII, pOper, and MCC. Pseudo-stimulation resulted in greater activation in the precuneus compared with genuine stimulation. Additionally, genuine stimulation induced greater deactivation in some default-mode regions, including the MTG and parahippocampus. (**b**) The inverse of the PSE (an estimate of tactile sensitivity) was positively correlated with differences in the parameter coefficient value in the bilateral SI, ipsilateral PCC, mPFC and parahippocampus, but negatively correlated with the bilateral aIns.

We found several differences in brain responses between genuine acupuncture stimulation and pseudo-stimulation (note that clusters with significant positive Z-scores may arise from either greater activation with genuine acupuncture stimulation or greater deactivation with pseudo-stimulation). Genuine acupuncture stimulation induced greater activation in the contralateral posterior insula (pIns), SMG, SII and bilateral posterior operculum (pOper) and mid-cingulate cortex (MCC), whereas pseudo-stimulation produced greater activation in the bilateral precuneus. Furthermore, genuine acupuncture stimulation also induced greater deactivation in some default-mode regions, including the MTG and parahippocampus (see Table 5 and Fig. 4a). The anterior part of the insulo-cingulate network was similarly activated in response to both sets of stimuli, whereas the posterior part was more strongly related to genuine acupuncture stimulation.

### Individual differences in differential brain responses to genuine acupuncture compared with pseudo-stimulation covariance with tactile sensitivity

The PSE of the two-point discrimination task was measured for each participant prior to the fMRI sessions. The inverse of the PSE can be used as an estimate of tactile sensitivity. We investigated the correlation between the parameter coefficient value (genuine acupuncture - pseudo-stimulation) and the inverse of the PSE. The tactile sensitivity was significantly and positively correlated with differences in the parameter coefficient value in the bilateral SI (contralateral: *r* = 0.913, p < 0.001; ipsilateral: *r* = 0.807, *p* = 0.002), ipsilateral PCC (*r* = 0.931, *p* < 0.001), mPFC (*r* = 0.913, *p* < 0.001) and parahippocampus (*r* = 0.761, *p* < 0.001) – these regions are part of the DMN, as well as primary sensory processing regions. Negatively correlated regions were found to include the bilateral aIns (contralateral: *r* = −0.881, *p* < 0.001; ipsilateral: *r* = −0.830, *p* = 0.001), which represents subjective feeling (see Fig. 4b, Supplementary table S2).

## Discussion

Genuine acupuncture stimulated several networks of brain activation, including the SI/SII, ACC (pregenual anterior, dorsal anterior and middle), insula (anterior, mid, posterior and operculum), dlPFC, and premotor area, as well as brain deactivation in the medial prefrontal cortex, posterior cingulate cortex, inferior parietal cortex and the parahippocampus (see Table 2 and Fig. 2a). Furthermore, pseudo-stimulation produced similar brain activation in the saliency network and deactivation in the DMN (see Table 3 and Fig. 2b). In both sessions, participants were required to discriminate which points were stimulated in a 2AFC task under enhanced bodily attention around the acupoint. Thus, we assumed that the brain response patterns to pseudo-stimulation might be strongly associated with accentuated bodily attention in certain parts of the body. Via a conjunction analysis between the two conditions, we demonstrated a common brain network involved in the top-down modulation of acupuncture stimulation triggered by bodily attention.

The main findings of the conjunction analysis included brain activation in the anterior insula and the ACC. The anterior insula and the ACC are functionally and anatomically entangled areas^27^,^28^,^29^ that play a crucial role in saliency and attention^30^. Increased activity of the ACC has been observed particularly in cognitively challenging situations where attention is needed; in these situations, the activity of the ACC was correlated with changes in the autonomic nervous system^31^. The anterior insula is well known for its integrative role in afferent and visceral information and representation of subjective feeling in diverse domains, including pain perception^32^. Activation of the ACC also triggers the endogenous analgesia system and modulates sensory transmission at the level of the spinal cord via descending inhibitory modulation^33^. Several studies support this modulatory function of the autonomic nervous system^34^,^35^,^36^. Reduced pain ratings followed by meditation were associated with increased activity in the ACC and anterior insula^15^. Compared with control, meditation experts found increased activity in the anterior insula and ACC in response to pain, which in turn was associated with reduced pain ratings and faster neural habituation of the amygdala^37^. Although our experiments were not designed to observe analgesic effects or other clinical outcomes, we could expect that the recruitment of the anterior insula and ACC triggered by bodily attention in acupuncture stimulation has the potential to be linked to the modulatory function of pain or the autonomic nervous system.

We also demonstrated that genuine acupuncture stimulation resulted in greater activity in the bilateral posterior insula/operculum in the MCC and in the contralateral secondary somatosensory processing areas, compared with pseudo-stimulation (see Table 5 and Fig. 4a). Because there were no differences in the external situations or the tasks (with the exception of the somatosensory stimulation), we may assume that differences in brain response between genuine acupuncture and pseudo-stimulation derive from the external physical afferent somatosensory signal. We demonstrated that genuine stimulation resulted in greater brain activation in the posterior insula than in the anterior insula, as well as in the MCC than in the ACC. It is well known that anticipation of pain engages the anterior insula, whereas the actual experience of pain or somatosensory signal engages the mid and posterior insula^32^,^38^,^39^. Greater engagement to direct afferent somatosensory signals in the posterior insula is supported by its connections with structures including the thalamus and basal ganglia^38^,^40^,^41^. The posterior insula receives afferent projections from the lamina I spinothalamocortical pathway, carrying nociceptive, thermal, and other interoceptive information^38^, and it also receives signals from un-myelinated (C) afferents^40^. Thus, it is expected that greater activation in the posterior insula and MCC with genuine acupuncture stimulation might be associated with greater engagement with afferent somatosensory signals from manual acupuncture.

The conjunction map indicates that both genuine- and pseudo stimuli produced deactivations of the DMN, including inferior parietal lobe, posterior cingulate cortex, medial temporal gyrus, parahippocampus, and medial prefrontal cortex. These findings were consistent with previous studies in which deactivations in the DMN were induced by afferent somatosensory signals from genuine acupuncture stimulation^5^. It is well known that both medial prefrontal cortex and PCC are involved in self-referential processing, which support self-reflection about internal thoughts and feelings in the absence of external stimulus processing^42^,^43^. Moreover, the DMN was deactivated during several types of meditation, such as focused attention on the breath, loving-kindness, and choiceless awareness in experienced meditators^44^. Recently, real-time feedback during a focused attention task found activation of the PCC during mind-wondering and deactivation during meditation^45^. Hence, we may assume that the deactivations of the DMN even during pseudo-stimulation in this study might be derived from enhanced bodily attention to certain parts of the body.

In this work, there were individual differences in tactile sensitivity around the acupoints in the forearm in the two-point discrimination task. We demonstrated that increased brain activations in response to genuine stimulation were correlated with increased tactile sensitivity in the SI, mPFC, PCC, and parahippocampus (see Fig. 4b). As an early tactile processing area, the SI plays a significant role in the signal processing of afferent sensory input. Brain activation in the SI was found in response to genuine stimulation, but not in response to pseudo-stimulation. We may expect that greater activation of the SI in response to greater tactile sensitivity will be associated with greater signal processing abilities of afferent somatosensory during genuine stimulation. In contrast, greater brain activation in response to pseudo-stimulation was correlated with greater tactile sensitivity in the anterior insula (see Fig. 4b). With pseudo-stimulation, participants were asked to discriminate the sensory information under the circumstances of no external stimulation. A recent neuroimaging study found that anticipatory processes using contextual information are associated with anterior insula activity, and that this also strongly influences pain perception^46^. The anterior insula integrates anticipation and actual sensory input 46. Since participants with greater tactile sensitivity were better able to detect discrepancies between expected sensation and the afferent signal, we may conjecture that greater brain activation in response to pseudo-stimulation is correlated with greater brain activity in the anterior insula. Due to the exploratory nature of this study, an alpha level of p < 0.001 was chosen, and corrections for multiple comparisons were not implemented in the correlation analysis between the sensitivity of stimuli and difference of genuine-pseudo brain responses in this study.

There were some limitations to this study. Because we aimed to facilitate bodily attention to a specific body point, even when there was no sensation (i.e., with pseudo-stimulation in session 2), we performed the two sessions in a fixed order, i.e., genuine stimulation first and pseudo-stimulation second. Although these conditioning methods allowed the participants to focus bodily attention on the pseudo-stimulation session, we cannot rule out the possibility of habituation or sensitization in the response to acupuncture stimulation^47^,^48^. Second, since the participants were told that they received electro-acupuncture in the pseudo-stimulation session, the brain response to bodily attention during pseudo-stimulation might be related to expectation or placebo effects. Because we informed the participants that they would receive one of two *active* treatments (manual or electro-acupuncture stimulation), the conjunction analysis and the differential analysis were not able to rule out other top-down components of the acupuncture treatment, such as the expectation effect. It is possible that non-penetrating placebo needles or sham point stimulation may not only exert an expectation factor but also elicit focused bodily attention around the acupoints^49^,^50^. Because we did not investigate the role of bodily attention during placebo acupuncture, we were unable to distinguish the components of bodily attention from other tactile stimulation. However, our study clearly demonstrates common and differential brain responses between external physical stimulation and internal bodily attention components during acupuncture stimulation. As we did not implement a fully-crossed 2 × 2 (Bodily attention × Stimulation) factorial design with a control condition for bodily attention, we cannot fully claim that there is no possible interaction between bodily attention and the sensation of external physical stimuli. Further studies are necessary to explore neural correlates of physical stimulation apart from the existence of bodily attention. Last but not least, participants were required to discriminate sensory information from two different acupoints: HT7 (ulnar nerve innervation) and PC6 (median nerve innervation), alternatively in both genuine acupuncture and with pseudo-stimulation. Concerns may arise regarding point-specific brain responses between these two points; however, there were no significant differences in brain responses between the two different acupoints using a typical general linear model. Instead, we plan to analyze different patterns of brain activations between the acupoints using multi-voxel pattern analysis as part of future work.

In summary, we have investigated the brain regions involved in top-down modulation (conjunction analysis between genuine acupuncture and pseudo-stimulation) and bottom-up modulation (difference between genuine acupuncture and pseudo-stimulation) through dissociation of the bodily attention component. We demonstrated that enhanced bodily attention around a certain part of the body resulted in brain activation in the salient interoceptive-autonomic network, especially the insula, anterior cingulate cortex, as well as deactivation in the DMN, especially the medial prefrontal cortex, posterior cingulate cortex, inferior parietal cortex and the parahippocampus, with both genuine acupuncture and pseudo-stimulation. We also found that external physical stimulation resulted in greater brain activation in the posterior insula, posterior operculum and caudal part of anterior cingulate cortex. These findings suggest that the components of enhanced bodily attention around the acupoint are significant in the neurophysiological response to acupuncture stimulation.

## Additional Information

**How to cite this article**: Jung, W.-M. *et al.* Cortical Activation Patterns of Bodily Attention triggered by Acupuncture Stimulation. *Sci. Rep.* **5**, 12455; doi: 10.1038/srep12455 (2015).

## Supplementary Material

Supplementary Information

## Figures and Tables

**Table 1:** Behavioral results of genuine acupuncture and pseudo-stimulation.

Participant	PSE (mm)	Genuine acupuncture			Pseudo-stimulation		
		Rate of correct estimation	Intensity (PC6)	Intensity (HT7)	Rate of response	Intensity (PC6)	Intensity (HT7)
S01	24.98	1.00	3.3 ± 0.48	3.1 ± 0.56	0.55	1.6 ± 0.51	1.8 ± 0.44
S02	37.06	1.00	2.3 ± 0.94	2.9 ± 0.73	1.00	1.0 ± 0.00	1.0 ± 0.27
S03	34.05	1.00	1.6 ± 0.69	2.2 ± 0.63	1.00	1.1 ± 0.37	1.0 ± 0.28
S04	37.78	1.00	2.4 ± 0.51	3.2 ± 0.42	0.85	1.3 ± 0.48	1.2 ± 0.42
S05	26.79	0.95	2.8 ± 1.03	2.7 ± 0.67	0.25	1.1 ± 0.33	1.1 ± 0.40
S06	23.00	1.00	2.0 ± 0.66	2.4 ± 0.51	1.00	1.3 ± 0.48	1.4 ± 0.53
S07	32.07	1.00	2.9 ± 0.87	1.7 ± 0.82	0.95	1.0 ± 0.28	1.2 ± 0.46
S08	35.14	1.00	1.6 ± 0.51	3.8 ± 0.42	1.00	1.0 ± 0.00	1.0 ± 0.30
S09	38.14	0.90	2.4 ± 0.52	2.2 ± 1.31	1.00	1.0 ± 0.00	1.0 ± 0.00
S10	36.04	1.00	2.0 ± 0.66	2.8 ± 0.91	1.00	1.0 ± 0.00	1.0 ± 0.00
S11	25.95	1.00	3.5 ± 0.52	1.7 ± 0.67	1.00	1.3 ± 0.74	1.8 ± 0.71
S12	33.03	0.95	3.1 ± 0.87	3.4 ± 0.51	1.00	1.3 ± 0.49	1.0 ± 0.00
S13	34.05	1.00	2.8 ± 0.63	1.9 ± 0.87	1.00	1.0 ± 0.00	1.0 ± 0.00
S14	43.00	1.00	2.2 ± 0.66	3.1 ± 0.73	1.00	1.7 ± 1.21	1.6 ± 0.91
average	32.93 ± 5.79 (global PSE: 32.62)	0.986 ± 0.031	2.50 ± 0.89	2.65 ± 0.94	0.900 ± 0.224	1.24 ± 0.56	1.25 ± 0.51

**Table 2:** Summary of fMRI main effect map data for genuine acupuncture stimulation.

Genuine stimulation	Cluster peak z-score	Side	Brain region labels	Size (mm^2^)	*p*-Cluster	Coordinates (MNI)		
						X	Y	Z
Activation	8.36	R	vlPFC, aIns, pIns, operculum, SMA, SII, SMG, SPL	13295.9	<0.001	41	18	9
	7.57	R	ACC, preSMA	2921.2	<0.001	10	11	48
	7.45	L	aIns, Operculum, SMA, SII, SMG, SPL	9144.9	<0.001	−57	−24	22
	6.61	L	mACC, preSMA	1746.0	<0.001	−9	4	50
	5.13	L	fusiform area	878.8	<0.001	−8	−72	−1
	5.06	R	SI	436.5	0.006	17	−42	66
	4.97	R	fusiform area	1201.8	<0.001	6	−69	3
Deactivation	6.25	L	IPL	2453.3	<0.001	−44	−68	22
	5.83	R	IPL	1370.6	<0.001	44	−75	17
	5.78	L	Parahippocampus	972.8	<0.001	−31	−36	−15
	5.68	L	vmPFC, dlPFC	4100.6	<0.001	−8	56	−5
	5.22	L	MTG	2283.3	<0.001	−50	2	−29
	4.90	L	dPCC (BA23)	2587.5	<0.001	−8	−59	25
	4.33	R	vmPFC	844.6	<0.001	9	53	−3
	4.26	R	MI	535.0	<0.001	8	−39	62
	3.86	R	dPCC (BA23)	1362.8	<0.001	9	−54	60
	3.81	L	occipital lobe	717.8	<0.001	−19	−81	42
	3.46	R	occipital lobe	476.8	0.003	20	−75	44
	3.05	R	dlPFC	317.6	0.049	20	27	50

**Table 3:** Summary of fMRI main effect map data for pseudo-stimulation.

Pseudo Stimulation	Cluster peak z-score	Side	Brain region labels	Size (mm^2)	p-Cluster	Coordinates (MNI)		
						X	Y	Z
Activation	6.72	R	aIns, operculum, MI, SMA	4986.3	<0.001	30	27	7
	6.58	L	SMA	1261.0	<0.001	−27	−1	47
	6.27	L	ACC	1320.7	<0.001	−12	24	33
	5.97	L	SII, SMA, SPL, precuneus	5127.4	<0.001	−29	−48	39
	5.61	L	aIns, operculum, MI	2859.4	<0.001	−29	22	−3
	5.59	R	ACC, preSMA	1700.4	<0.001	11	16	41
	5.29	R	SPL	1144.8	<0.001	31	−48	46
	5.01	R	Precuneus	752.7	<0.001	7	−72	45
	4.77	L	fusiform area	1570.1	<0.001	−14	−90	2
	4.73	R	SII (BA43)	717.1	<0.001	62	−16	23
	3.86	R	fusiform area	713.6	<0.001	28	−68	−4
	3.71	R	vlPFC	590.9	0.001	24	43	−10
	2.44	R	occipital lobe	421.0	0.017	17	−99	−3
**Deactivation**	4.78	R	dPCC (BA23)	644.3	<0.001	6	−50	21
	4.51	R	IPL	467.9	0.009	50	−62	23
	4.46	L	dPCC (BA23)	1415.8	<0.001	−8	−57	10
	4.29	L	MTG	641.4	0.001	−57	−11	−22
	4.19	L	IPL	1292.8	<0.001	−43	−67	24
	4.06	L	vmPFC, dlPFC	2602.8	<0.001	−21	25	36
	3.53	R	MTG	432.8	0.015	54	−2	−31
	3.47	R	dlPFC	733.1	<0.001	9	56	15
	3.05	R	SI	972.4	<0.001	35	−20	50

**Table 4:** Summary of fMRI conjunction map data between genuine acupuncture and pseudo-stimulation.

Conjunction Map	Cluster peak z-score	Side	Brain region labels	Size (mm^2^)	*p*-Cluster	Coordinates (MNI)		
						X	Y	Z
Activation	6.72	R	aIns, pIns, MI	4540.6	<0.001	30	27	7
	5.64	L	ACC	983.3	<0.001	−11	14	44
	5.59	R	ACC, preSMA	1519.2	<0.001	11	16	41
	5.51	L	aIns, Operculum, MI	2475.7	<0.001	−29	23	−2
	4.92	L	SII, SMA, SPL	2927.4	<0.001	−54	−21	30
	4.79	L	preSMA	199.1	<0.001	−8	0	66
	4.73	R	SII (BA43)	717.1	<0.001	62	−16	23
	4.66	L	SMA	854.7	<0.001	−32	−5	43
	4.47	L	vPCC (BA24)	229.3	<0.001	−5	−24	27
	3.74	R	vPCC (BA24)	284.2	<0.001	5	−25	27
	3.51	L	fusiform area	664.4	<0.001	−10	−79	−4
	3.45	R	SPL	747.5	<0.001	31	−47	41
	3.40	L	occipital lobe	102.6	0.009	−12	−85	1
	3.29	R	SII (BA40)	350.1	<0.001	46	−32	39
	3.13	R	fusiform area	516.6	<0.001	5	−74	3
	3.01	R	dlPFC(BA10)	219.4	<0.001	36	50	10
	2.93	R	dlPFC (BA46)	124.6	0.002	38	39	17
	2.87	R	Precuneus	196.4	<0.001	14	−67	37
	2.75	R	SMG (BA42)	122.7	0.002	52	−38	21
**Deactivation**	4.51	R	IPL	444.1	<0.001	50	−62	23
	4.19	L	IPL	1253.9	<0.001	−43	−67	24
	4.16	L	MTG	546.1	<0.001	−56	−10	−23
	4.02	L	vmPFC, dlPFC	2484.4	<0.001	−21	25	36
	3.97	L	dPCC (BA23)	1369.8	<0.001	−5	−61	22
	3.46	R	dPCC (BA23)	623.0	<0.001	9	−56	31
	3.28	R	vmPFC	157.7	<0.001	11	51	−7
	3.20	L	parahippocampus	186.2	<0.001	−30	−26	−23
	2.95	R	SI	165.0	<0.001	43	−19	49
	2.51	R	dlPFC (BA9)	131.2	0.001	24	30	34
	2.48	R	dmPFC	107.6	0.006	9	50	15

**Table 5:** Summary of fMRI difference map data between genuine acupuncture and pseudo-stimulation.

Difference Map (Genuine - Pseudo Stimulation)	Cluster peak z-score	Side	Brain region labels	Size (mm^2^)	*p*-Cluster	Coordinates (MNI)		
						X	Y	Z
Activation	7.53	R	pIns, SII, SMG	5174.0	<0.001	63	−13	19
	5.91	L	pIns, SII, SMG	1828.2	<0.001	−55	−25	21
	5.37	R	MCC	1159.5	<0.001	4	−9	37
	4.93	R	SI	880.3	<0.001	25	−43	57
**Deactivation**	3.67	L	MTG	744.6	<0.001	−50	4	−15
	3.46	R	precuneus	547.1	0.003	9	−64	47
	3.42	L	precuneus	894.6	<0.001	−7	−70	49
