# Supplementary material for: Cortical Activation Patterns of Bodily Attention triggered by Acupuncture Stimulation
Source: Sci Rep. 2015 Jul 27;5:12455. doi: 10.1038/srep12455 (PMC4515634; doi:10.1038/srep12455)
Supplement: Supplementary Information [file srep12455-s1.doc]

**Supplementary information**

**Cortical Activation Patterns of Bodily Attention triggered by Acupuncture Stimulation**

**Won-Mo Jung, In-Seon Lee, Christian Wallraven,**

**Yeon-Hee Ryu, Hi-Joon Park, Younbyoung Chae**

**Supplementary Material (for the volume-based analysis)**

For the analysis of the regions excluded in the surface-based analysis, a volumetric analysis was run using AFNI software (NIMH, USA). The volume-based analysis used the same parameters as the surface-based analysis. Instead of the surface model, which individually registered the reconstructed surface, equivalently realigned functional images were transformed to Talairach space, and blurred with a Gaussian kernel of full-width half-maximum (FWHM) value of 4 mm. A time-series statistical analysis was performed to explore brain areas showing significant changes of BOLD signals to genuine stimulation or to pseudo stimulation with reference to the baseline, using a general linear model of AFNI’s 3dDeconvolve. One-sample t-tests across the individual contrast images were used to detect significant activation on a group level. The significance level for group data was set at a *t*-value threshold of 2 and multiple comparison correction was conducted (*p* < 0.05) using a Monte Carlo simulation (10,000 iterations).

| **Supplementary Table 1. Summary of fMRI main effect map data for genuine acupuncture stimulation and pseudo-stimulation in the subcortex regions and the cerebellum.** | | | | | | | | |
| --- | --- | --- | --- | --- | --- | --- | --- | --- |
| Main Effects | Cluster peak  z-score | Side | Brain region labels | Size  (voxel) | p-Cluster (corrected) | Coordinates  (MNI) | | |
|  |  |  |  |  |  | X | Y | Z |
| Genuine  Stimulation | 4.93 | R  (extending to left also) | thalamus, putamen, caudate | 101 | <0.001 | -11 | 12 | 7 |
|  | 4.74 | R | Lobule VIIIa, VIIb | 42 | <0.001 | 20 | -60 | -45 |
|  | 4.71 | R | Lobule VI, VIIa Crus I | 34 | <0.001 | 30 | -50 | -25 |
|  | 4.49 | L | Lobule VIIb, VIIIa | 27 | <0.001 | -19 | -64 | -46 |
| Pseudo  Stimulation | 4.84 | R | thalamus, putamen, caudate | 55 | <0.001 | 12 | 13 | 5 |
|  | 4.87 | L | thalamus, putamen, caudate | 48 | <0.001 | -13 | 13 | 5 |

| Supplementary Table 2. Summary of fMRI covariates map data of two main effects (genuine, pseudo) and difference (genuine-pseudo) to inverse of PSE. | | | | | | | | |
| --- | --- | --- | --- | --- | --- | --- | --- | --- |
| Covariates Map to Main Effects | Cluster peak  z-score | Side | Brain region labels | Size  (voxels) | p-Value (uncorrected) | Coordinates  (MNI) | | |
|  |  |  |  |  |  | X | Y | Z |
| Genuine acupuncture  stimulation | 4.578 | R | SPL | 17.0 | <0.001 | 22 | -51 | 60 |
|  | 4.191 | R | PCC | 13.3 | <0.001 | 13 | -24 | 35 |
|  | 3.572 | R | MI | 6.1 | <0.001 | 17 | -19 | 64 |
|  | 4.214 | L | SPL | 13.9 | <0.001 | -18 | -74 | 36 |
|  | 3.795 | L | MI | 11.6 | <0.001 | -23 | -14 | 59 |
|  | 3.725 | L | mOFC | 14.4 | <0.001 | -7 | 18 | -10 |
|  | 3.243 | L | SI | 10.8 | <0.001 | -32 | -26 | 46 |
|  | 3.219 | L | IPL | 15.9 | <0.001 | -28 | -75 | 16 |
| Pseudo - stimulation | -4.612 | R | lateral occipital area | 29.2 | <0.001 | 16 | -84 | 22 |
|  | -4.471 | R | lateral occipital area | 69.8 | <0.001 | 12 | -95 | 10 |
|  | 3.805 | R | parahippocampus | 15.6 | <0.001 | 25 | -24 | -15 |
|  | 4.115 | L | PCC | 20.7 | <0.001 | -2 | -14 | 27 |
|  | -3.378 | L | precuneus | 8.4 | <0.001 | -13 | -44 | 55 |
|  | -3.173 | L | SI | 14.8 | <0.001 | -5 | -36 | 58 |
| **Difference between**  **two main effects**  **(Genuine – Pseudo)** | -4.182 | R | aIns | 36.8 | <0.001 | 43.3 | 37.2 | -8.9 |
|  | 3.802 | R | SI | 42.3 | <0.001 | 29.5 | -38.6 | 49.0 |
|  | 4.827 | L | PCC | 52.6 | <0.001 | -12.0 | -41.2 | 45.6 |
|  | 3.675 | L | mPFC | 19.7 | <0.001 | -7.1 | 48.6 | -9.3 |
|  | 3.663 | L | SI | 15.7 | <0.001 | -20.1 | -36.9 | 57.8 |
|  | -3.499 | L | aIns | 18.1 | <0.001 | -30.7 | 19.4 | -2.7 |
|  | 3.351 | L | parahippocampal | 5.5 | <0.001 | -22.5 | -18.5 | -23.8 |
